# Evaluation of hypoglycemic, hypolipidemic and antioxidant effects in vivo of extracts hydroalcoholic of Yacon (Smallanthus sonchifolius)

**DOI:** 10.1186/1758-5996-7-S1-A64

**Published:** 2015-11-11

**Authors:** Patricia Martinez Oliveira, Patricia Maurer, Bruna Cocco Pilar, Ritiele Pinto Coelho, Angelica Aparecida da Costa Gullich, Vinicius Tejada Nunes, Jacqueline da Costa Escobar Piccoli, Vanusa Manfredini

**Affiliations:** 1 Universidade Federal do Pampa, Uruguaiana, Brazil

## Background

Cardiovascular diseases are the leading cause of death worldwide, and dyslipidemia a major risk factor. Feeding is recognized as the most important intervention in the prevention of diseases and plants are considered the best source of natural antioxidants. Yacon, a tuberous root, originates in the Andean region with fructooligosaccharides as the main reserve carbohydrate. In addition to these compounds, Yacon presents significant amount of polyphenols in both roots and leaves.

## Objective

Investigate the hypolipemic, hypoglycemic and antioxidant effects in vivo of extracts of leaf and root of Yacon.

## Materials and methods

Male Wistar rats were divided into: group 1 (normal control diet), group 2 (control calorie diet), group 3: oral suspension of simvastatin 10 mg/kg, group 4: Yacon leaf extract 20 mg/kg, group 5: Yacon leaf extract 40 mg/kg, group 6: Yacon leaf extract 20 mg/kg and simvastatin 10 mg/kg, group 7: Yacon leaf extract 40 mg/kg and simvastatin 10 mg/kg, group 8: Yacon root extract 20 mg/kg, G9: Yacon root extract 40 mg/kg, G10: Yacon root extract 20 mg/kg and simvastatin 10 mg/kg, G11: Yacon root extract 40 mg/kg and simvastatin 10 mg/kg. The formulations were administered once daily by gavage for 14 consecutive days. The hemograms and biochemistry parameters were determined by automated equipment. The oxidative parameters were measured using spectrophotometric methods.

## Results

The groups that received the Yacon extract showed improvement of glycemic and lipid profile. The hypercholesterolemic diet increased serum levels of creatine kinase, CK-MB and LDH, but the extract administration decreased the levels of these markers significantly when compared to the untreated group. Moreover, the extract, reduced lipid peroxidation, protein carbonylation and frequency of micronucleus induced by hypercholesterolemia and increase antioxidant defenses (CAT, SOD, GPx, GSH, vitamin C, polyphenols) in the blood. Moreover, supplementation of Yacon showed no hepatotoxic or nephrotoxic effect. The hypercholesterolemic diet increased the inflammatory process, evaluated through your markers, and extract administration has improved this parameter. Furthermore, supplementation with the root of Yacon controlled weight gain of animals.

## Conclusion

The results suggest that Yacon extract showed a hypoglycemic, hypolipidemic and antioxidant activity, possibly due to its high content of phenolic compounds.

